# A Glimpse Into Rarity: A Phenomenal Case of Autoimmune Retinopathy in a Young Woman

**DOI:** 10.7759/cureus.71387

**Published:** 2024-10-13

**Authors:** Nanditha Nair, Anusha Venkatraman, Renu Magdum, Ozukhil Radhakrishnan

**Affiliations:** 1 Ophthalmology, Dr. D.Y. Patil Medical College, Hospital and Research Centre, Dr. D.Y. Patil Vidyapeeth (Deemed to be University), Pune, IND; 2 Ophthalmology, Sankara Eye Hospital, Coimbatore, IND

**Keywords:** autoantibodies, autoimmune retinopathy, immunosuppressants, nyctalopia, photopsia

## Abstract

Autoimmune retinopathy (AIR) is a rare retinal disorder that causes a gradual loss of vision due to autoantibodies targeting retinal antigens, leading to photoreceptor degeneration. Early diagnosis and timely intervention are critical for preserving visual function in affected patients. Over the course of a year, a 30-year-old woman had bilateral, abrupt, painless, progressive diminution of vision, nyctalopia, photopsia, and a restricted peripheral field of vision. No family history of night blindness was found. A diagnosis of AIR was suggested by the clinical examination, fundus fluorescein angiography (FFA), negative electroretinogram (ERG), and the short duration and quick progression of symptoms. A differential diagnosis of photoreceptor dystrophy was considered. Immunosuppressants and oral corticosteroids were started as treatment. Within a month of follow-up, the patient's vision had significantly improved. Despite the poor guarded prognosis of AIR, a favorable visual outcome was achieved through early detection and treatment with immunosuppressants.

## Introduction

Autoimmune retinopathy (AIR) is a rare immune-mediated retinal disorder characterized by the production of autoantibodies that target retinal antigens, leading to progressive visual impairment [1]. It can occur either in isolation or in association with systemic autoimmune diseases such as systemic lupus erythematosus, rheumatoid arthritis, or Sjögren's syndrome [2-4]. AIR is broadly categorized into two types: paraneoplastic AIR (pAIR), associated with malignancies such as melanoma and small-cell lung carcinoma, and non-paraneoplastic AIR (npAIR), where no malignancy is present [1]. Despite its rarity, AIR represents a significant clinical entity due to its potential for irreversible vision loss.

Since AIR is a rare condition with limited data available, the exact prevalence of this disorder is unknown. Paraneoplastic forms are particularly rare, often affecting middle-aged to older adults with underlying malignancies, while non-paraneoplastic forms can affect individuals across a wider age range, regardless of their autoimmune disease history [5]. The true incidence of AIR is likely underestimated due to its variable clinical presentation and the challenges associated with its diagnosis.

The pathogenesis of AIR involves a breakdown in immune tolerance, leading to the production of autoantibodies that target key retinal antigens such as recoverin, S-arrestin, or transducin. These autoantibodies induce inflammation, disrupt photoreceptor function, and lead to retinal degeneration. In pAIR, the immune response is triggered by cross-reactivity between retinal antigens and tumor-associated antigens, whereas in npAIR, the mechanism is more likely related to systemic autoimmune dysregulation [1,5]. Despite these differences in etiology, both forms share a common pathway of immune-mediated retinal damage, resulting in visual impairment.

Clinically, AIR presents with bilateral, painless, progressive vision loss, often accompanied by photopsias, nyctalopia, and visual field defects [6]. The onset of symptoms is typically insidious, and initial fundoscopic exams may appear normal, leading to delayed diagnosis. Over time, retinal findings such as vascular attenuation, optic disc pallor, and photoreceptor loss become more evident. The clinical course of AIR is variable, with some patients experiencing rapid deterioration of vision while others show a more gradual progression [7,8].

The differential diagnosis for AIR includes several conditions that cause progressive vision loss, such as retinitis pigmentosa (RP), cancer-associated retinopathy (CAR), melanoma-associated retinopathy (MAR), inflammatory retinal diseases like uveitis, and inherited retinal dystrophies. Differentiating AIR from these conditions can be challenging due to overlapping clinical features, necessitating thorough clinical evaluation and specific diagnostic tests. Diagnosis is particularly difficult because AIR is rare, and early signs are often nonspecific. Key diagnostic tools include electroretinography (ERG), which typically shows reduced responses indicating impaired photoreceptor function; optical coherence tomography (OCT), which may reveal retinal thinning and loss of photoreceptor layers; and fluorescein angiography (FFA), which can detect vascular leakage and abnormalities [9]. Additionally, serological tests for antiretinal antibodies (ARAs) play a crucial role in supporting the diagnosis, although their presence alone is insufficient and must be interpreted in conjunction with clinical findings [5].

Management of AIR focuses on immunosuppressive therapy to control the autoimmune response and prevent further retinal damage. Treatment usually begins with high-dose corticosteroids, followed by steroid-sparing agents such as azathioprine, methotrexate, or mycophenolate mofetil. In more refractory cases, therapies like intravenous immunoglobulin (IVIG) or plasmapheresis may be used. There is no standardized treatment protocol, and the response to therapy is variable. While some patients achieve stabilization of their condition, others may continue to experience progressive vision loss despite treatment [1,5].

Complications associated with AIR are primarily related to delayed diagnosis and treatment, which can result in irreversible retinal damage and permanent vision loss. Long-term use of immunosuppressive therapy also carries the risk of side effects, including increased susceptibility to infections and other systemic complications [10]. Close monitoring and individualized treatment plans are essential to mitigate these risks and optimize patient outcomes.

This study presents a rare case of npAIR, shedding light on the diagnostic complexities and therapeutic challenges associated with this condition.

## Case presentation

A 30-year-old woman presented with bilateral, abrupt, painless, progressive diminution of distant and near vision, nyctalopia, and photopsia. She had received prior treatment with systemic antiviral drugs and steroids elsewhere for the same but had not experienced any significant improvement. The patient was born into a consanguineous marriage, but there is no family history of night blindness, autoimmune disease, or neoplastic disease. By diet, she is not a vegetarian. The systemic and general examinations were both normal. The serology came back negative, and all regular blood parameters, including serum homocysteine and vitamin B12, were within normal ranges (Table 1).

**Table 1:** Routine blood investigations. SGOT: serum glutamic-oxaloacetic transaminase; SGPT: serum glutamic pyruvic transaminase; ALP: alkaline phosphatase; T3: triiodothyronine; T4: thyroxine; TSH: thyroid-stimulating hormone; ESR: erythrocyte; RBS: random blood sugar.

Parameters	Result	Reference values
Hemoglobin	11 g/dL	12-16 g/dL
Total leucocyte count	9,600/μL	4,000-10,000/μL
Platelet count	2,32,000/μL	1,50,000-4,10,000/μL
Serum urea	38 mg/dL	17-49 mg/dL
Serum creatinine	0.62 mg/dL	0.6-1.35 mg/dL
Serum total bilirubin	1.1 mg/dL	0.2-1.2 mg/dL
Conjugated bilirubin	0.3 mg/dL	0.2-0.3 mg/dL
Unconjugated bilirubin	0.8 mg/dL	0.1-1 mg/dL
SGOT	32 IU/L	8-48 IU/L
SGPT	40 IU/L	7-55 IU/L
ALP	78 IU/L	35-104 IU/L
Serum sodium	138 mmol/L	136-145 mmol/L
Serum potassium	3.6 mmol/L	3.5-5.1 mmol/L
ESR	15 mm/h	Up to 20 mm/h
RBS	110 mg/dL	Up to 140 mg/dL
HIV antibody	Negative	-
Hepatitis B antibody	Negative	-
Hepatitis C antibody	Negative	-
T3	0.7 ng/mL	0.64-1.52 ng/mL
T4	5.87 ng/mL	4.87-11.72 ng/mL
TSH	3.93 ng/mL	0.35-4.94 ng/mL
Vitamin B12 levels	408 pg/mL	190-950 pg/mL
Serum homocysteine	6.2 mmol/L	5-15 mmol/L

**Figure 1:**
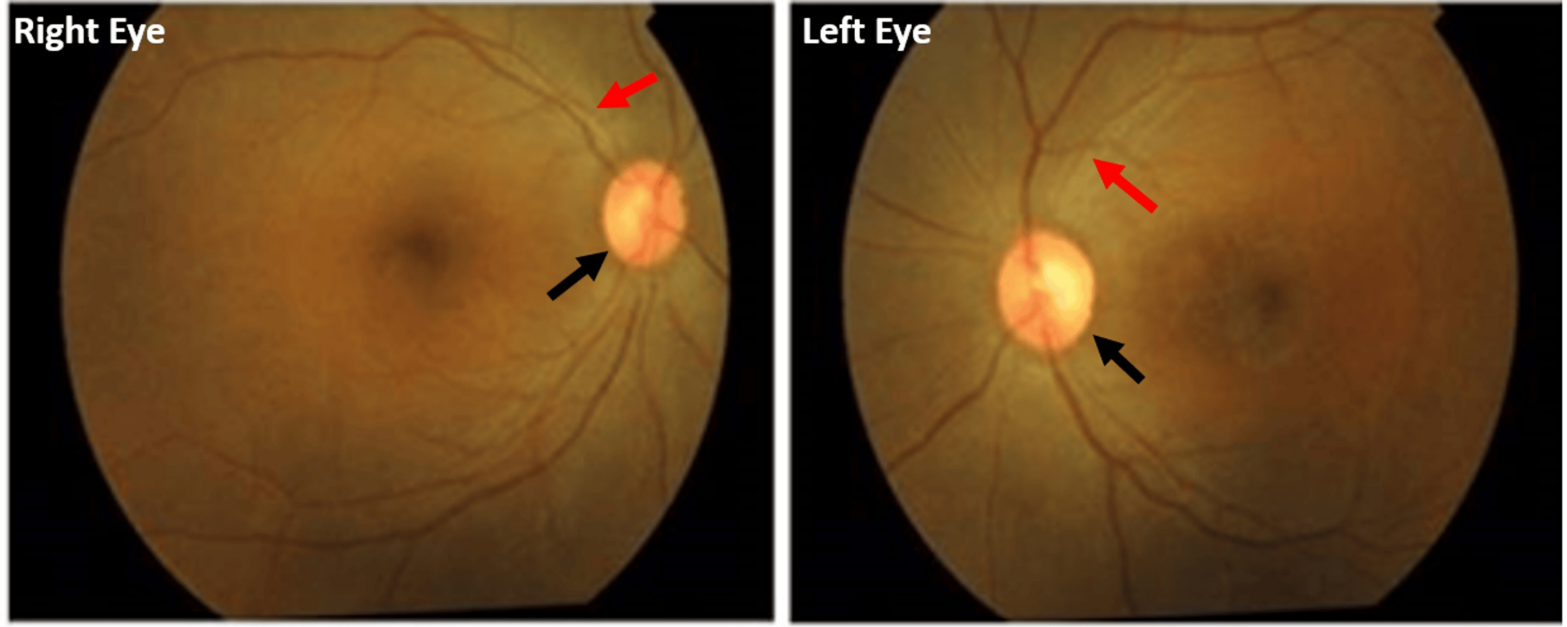
Color fundus photograph of both eyes showing mild disc pallor (black arrow), attenuated vessels (red arrow), and dull foveolar reflex.

On ocular examination, best-corrected visual acuity (BCVA) was 3/60 in the right eye and 2/60 in the left eye. Both eyes had impaired color vision and N24 near vision. Pupillary examination showed a sluggish reaction to light in both eyes. Fundus examination demonstrated a mild disc pallor, attenuated vessels, and dull foveolar reflex. Nevertheless, no bony spicules were seen (Figure 1).

**Figure 2:**
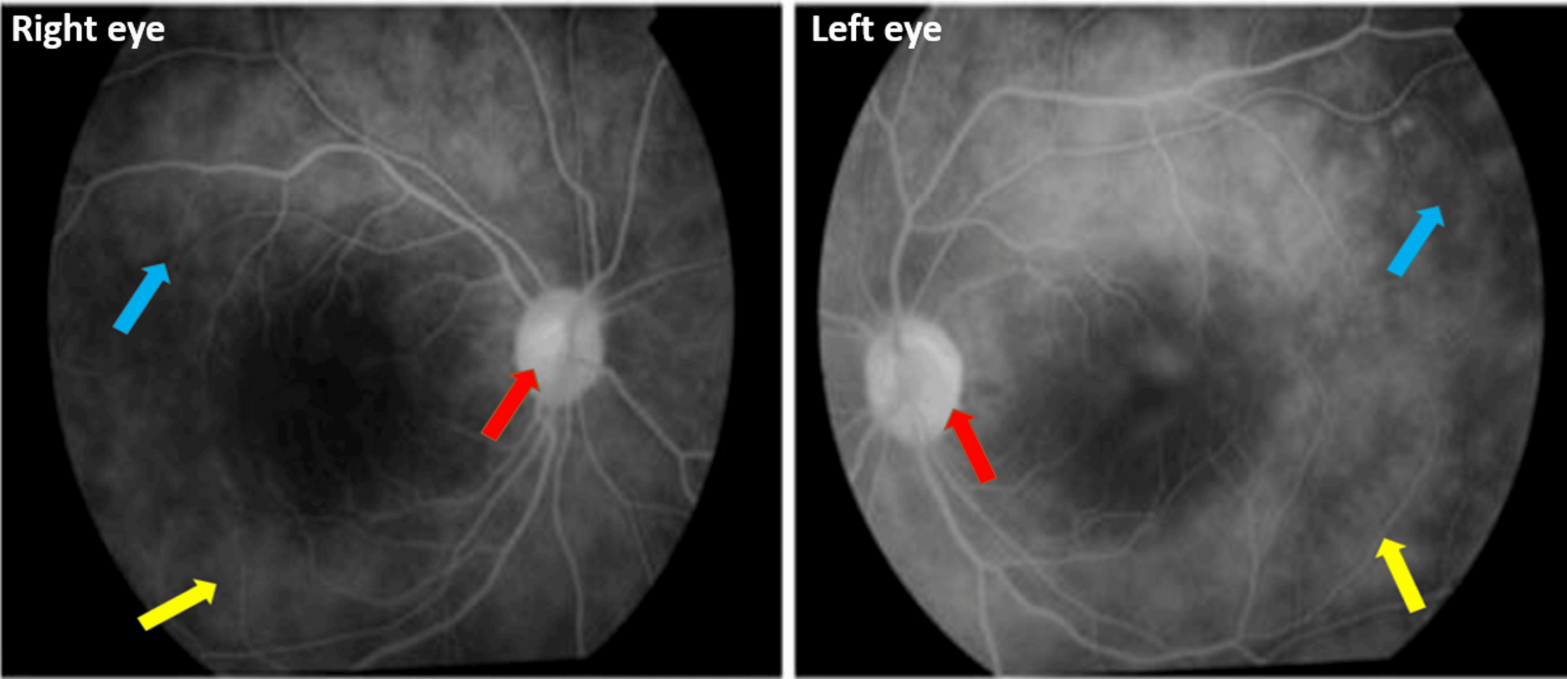
FFA of both eyes showing perivascular staining (blue arrow), pinpoint leakages (yellow arrow), and optic disc staining (red arrow). FFA: fundus fluorescein angiography.

Fundus fluorescein angiography (FFA) demonstrated multiple areas of pin point leakages, perivascular staining, and disc staining in the late phase (Figure 2).

**Figure 3:**
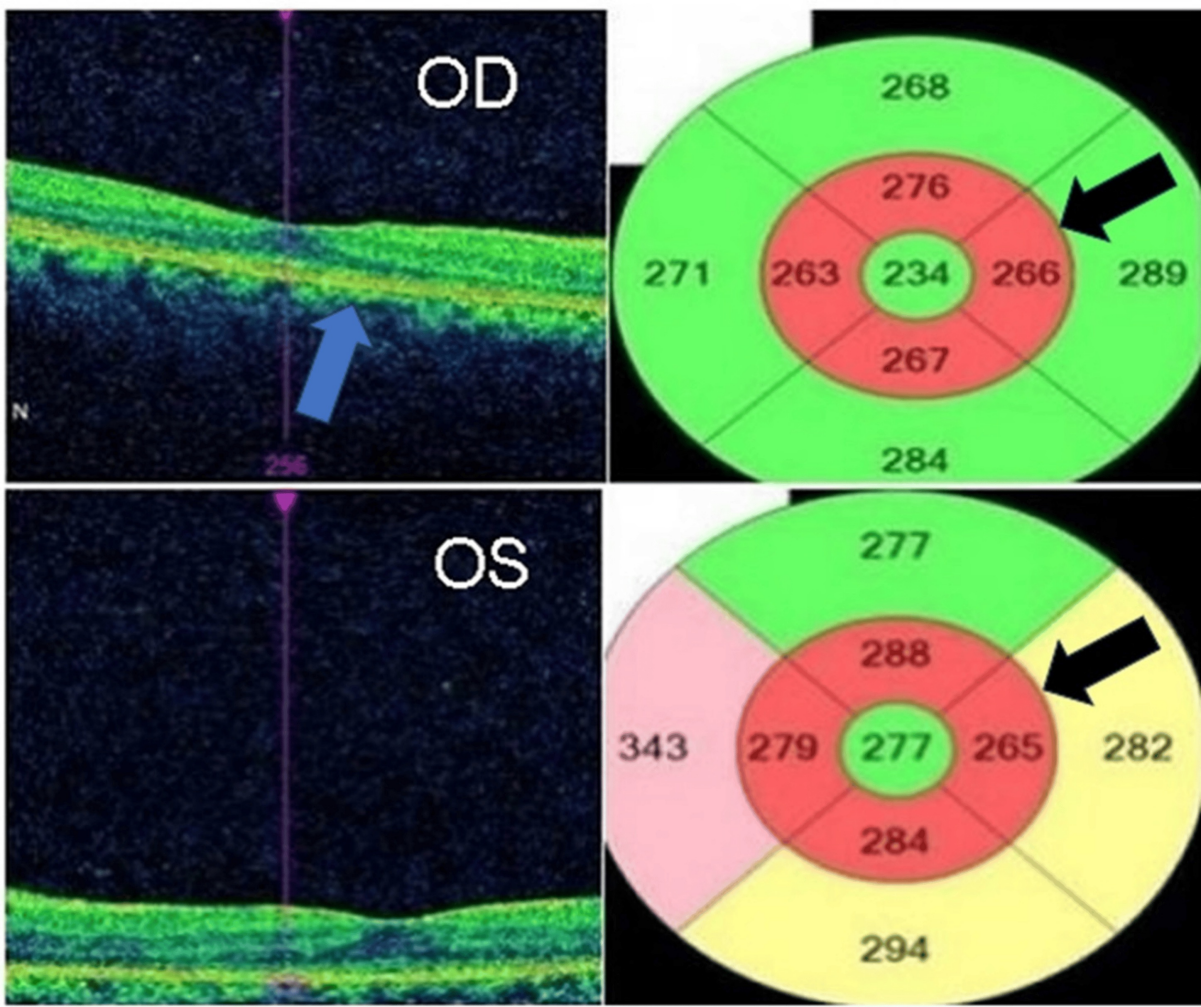
OCT (left) showing granularity (blue arrow) in the photoreceptor layer along with corresponding macular thickness map (right) revealing parafoveal thinning (black arrow). OCT: optical coherence tomography.

The extent of retinal damage was evaluated through OCT and quantified through a macular thickness map. OCT and macular thickness mapping revealed significant perifoveal thinning and granularity in the photoreceptor layer of both eyes, suggesting a generalized loss of both the inner and outer segments of the photoreceptors, a hallmark of retinal degeneration often associated with AIR (Figure 3).

The results of the PET scan and further blood tests were normal, barring out neoplastic diseases, or metastatic tumors (METS). With photoreceptor dystrophy as a differential diagnosis, npAIR was tentatively diagnosed. Treatment was initiated with oral prednisolone 50 mg once daily and oral azathioprine 50 mg twice daily after consultation with the physician. Her near vision improved to N10, and her BCVA improved to 6/60 after a month, which she maintained until the six-month follow-up. Throughout the course of the therapy, the patient was maintained on 50 mg of azathioprine daily while the steroids were gradually tapered off.

## Discussion

AIR is classified as paraneoplastic AIR (pAIR) and non-paraneoplastic AIR (npAIR). It is referred to as MAR when associated with melanoma and CAR when related to other cancers [6]. The precise prevalence of AIR remains undetermined.

npAIR has a female predominance (63%-66%), with the average age at diagnosis being 65 years [5]. A history of autoimmune disease, mostly hypothyroidism, may also be elicited in some patients. However, our patient was only 30 years old and had no such associations, making it a very rare presentation.

Scotomas, a visual field defect, photoreceptor malfunction, and abruptly progressive, painless vision impairment are common symptoms of AIR, which is typically bilateral [5]. The majority of patients initially show no abnormalities on fundus examination; however, in later stages, subtle abnormalities in the retina may be noted [6]. Our patient had subacute vision loss, and OCT, along with macular thickness mapping [11], indicated vascular attenuation in the fundus, granularity in the photoreceptor layer with parafoveal thinning, and an electronegative electroretinogram (ERG) that suggested the diagnosis of AIR.

ARAs are not considered to be a pathognomonic finding in AIR; nonetheless, utilizing a variety of imaging modalities in conjunction with the detection of circulating blood ARAs can help diagnose and evaluate the course of AIR [12,13]. We were unable to evaluate the presence of autoantibodies associated with AIR in our patient due to the lack of accessible testing facilities in our country. White dot syndromes, particularly acute zonal occult outer retinopathy (AZOOR), RP, cone-rod dystrophy, and various uveitic syndromes, are common differential diagnoses for AIR. We established a diagnosis of npAIR based on clinical findings and ancillary tests, including ERG, OCT, and FFA. Historically, management of AIR involved careful observation; however, the emerging treatment approach now includes systemic immunosuppression, notably the use of steroids. Other immunomodulatory therapies, such as intravenous immunoglobulin and plasmapheresis, have also been explored in the treatment of AIR [14,15].

Treatment options specifically aim to target the immune response triggered by autoantibodies related to AIR and to prevent further retinal degeneration and irreversible vision loss. While no single treatment modality has been definitively proven to be fully effective or capable of reversing photoreceptor damage, initiating therapy before irreversible damage occurs may facilitate vision recovery in AIR [16]. The notable visual improvement observed in our patient further supports the efficacy of oral steroids and immunosuppressants in managing this condition. Therefore, regardless of the patient's age at presentation, AIR should always be considered in the differential diagnosis of individuals experiencing sudden onset night blindness.

## Conclusions

AIR seldom occurs in young individuals. It continues to be a poorly understood immune-mediated disease that demands a personalized, systematic approach, taking into account all potential diagnoses. Early discovery and the initiation of immunosuppressive therapy in AIR can significantly improve the visual outcomes, despite a poor or dismal prognosis. Further research is required on attaining a standard management protocol so that appropriate therapy can be initiated for this highly challenging and uncertain disease. Research into AIR is ongoing, focusing on better understanding the underlying mechanisms, identifying specific biomarkers, and developing targeted therapies. As our knowledge expands, there is hope for improved diagnostic criteria and treatment options that can lead to better outcomes for those affected by this complex autoimmune condition.

## References

[1] Canamary AM Jr, Takahashi WY, Sallum JM (2018). Autoimmune retinopathy: a review. Int J Retina Vitreous.

[2] Wuthisiri W, Lai YH, Capasso J, Blidner M, Salz D, Kruger E, Levin AV (2017). Autoimmune retinopathy associated with systemic lupus erythematosus: a diagnostic dilemma. Taiwan J Ophthalmol.

[3] Negrini S, Emmi G, Greco M (2022). Sjögren's syndrome: a systemic autoimmune disease. Clin Exp Med.

[4] Glover K, Mishra D, Singh TRR (2021). Epidemiology of ocular manifestations in autoimmune disease. Front Immunol.

[5] Grange L, Dalal M, Nussenblatt RB, Sen HN (2013). Autoimmune retinopathy. Am J Ophthalmol.

[6] Heckenlively JR, Ferreyra HA (2008). Autoimmune retinopathy: a review and summary. Semin Immunopathol.

[7] Choi EY, Kim M, Adamus G, Koh HJ, Lee SC (2016). Non-paraneoplastic autoimmune retinopathy: the first case report in Korea. Yonsei Med J.

[8] Rahimy E, Sarraf D (2013). Paraneoplastic and non-paraneoplastic retinopathy and optic neuropathy: evaluation and management. Surv Ophthalmol.

[9] Raevis J, Etheridge T, Cleland S, Mititelu M (2020). Autoimmune retinopathy: findings and limitations from optical coherence tomography angiography. Int J Retina Vitreous.

[10] Ruiz R, Kirk AD (2015). Long-term toxicity of immunosuppressive therapy. Transplant Liver.

[11] Appukuttan B, Giridhar A, Gopalakrishnan M, Sivaprasad S (2014). Normative spectral domain optical coherence tomography data on macular and retinal nerve fiber layer thickness in Indians. Indian J Ophthalmol.

[12] Mrejen S, Khan S, Gallego-Pinazo R, Jampol LM, Yannuzzi LA (2014). Acute zonal occult outer retinopathy: a classification based on multimodal imaging. JAMA Ophthalmol.

[13] Smith-Hansen P, Gazieva L, Ilginis T (2016). AZOOR - a 10-year follow-up. Acta Ophthalmol.

[14] Fox AR, Gordon LK, Heckenlively JR (2016). Consensus on the diagnosis and management of nonparaneoplastic autoimmune retinopathy using a modified Delphi approach. Am J Ophthalmol.

[15] Braithwaite T, Vugler A, Tufail A (2012). Autoimmune retinopathy. Ophthalmologica.

[16] Chen SN, Yang CH, Yang CM (2015). Systemic corticosteroids therapy in the management of acute zonal occult outer retinopathy. J Ophthalmol.

